# Genome-Wide Identification and Expression Analysis of the *VILLIN* Gene Family in Soybean

**DOI:** 10.3390/plants12112101

**Published:** 2023-05-25

**Authors:** Yueqiong Zhou, Liangliang He, Shaoli Zhou, Qing Wu, Xuan Zhou, Yawen Mao, Baolin Zhao, Dongfa Wang, Weiyue Zhao, Ruoruo Wang, Huabin Hu, Jianghua Chen

**Affiliations:** 1 CAS Key Laboratory of Tropical Plant Resources and Sustainable Use, CAS Center for Excellence in Molecular Plant Sciences, Xishuangbanna Tropical Botanical Garden, Chinese Academy of Sciences, Kunming 650223, China; zhouyueqiong@xtbg.ac.cn (Y.Z.); liangl.he08@gmail.com (L.H.); zhoushaoli@xtbg.ac.cn (S.Z.); wuqing18@mails.ucas.ac.cn (Q.W.); 18383373840@163.com (X.Z.); maoyawen16@mails.ucas.ac.cn (Y.M.); zhaobl06@126.com (B.Z.); dongfaw0026@163.com (D.W.); zhaoweiyue18@163.com (W.Z.); ruozhu_w@163.com (R.W.); huhb@xtbg.ac.cn (H.H.); 2 University of Chinese Academy of Sciences, Beijing 101408, China; 3 College of Life Sciences, Division of Life Sciences and Medicine, University of Science and Technology of China, Hefei 230026, China; 4 Guizhou Institute of Biotechnology, Guizhou Academy of Agricultural Sciences, Guiyang 550006, China; 5 Institute of Biodiversity, School of Ecology and Environmental Science, Yunnan University, Kunming 650106, China

**Keywords:** soybean, VILLIN family, genome−wide analysis, expression profiling, abiotic stresses

## Abstract

The VILLIN (VLN) protein is an important regulator of the actin cytoskeleton, which orchestrates many developmental processes and participates in various biotic and abiotic responses in plants. Although the *VLN* gene family and their potential functions have been analyzed in several plants, knowledge of *VLN* genes in soybeans and legumes remains rather limited. In this study, a total of 35 VLNs were characterized from soybean and five related legumes. Combining with the VLN sequences from other nine land plants, we categorized the *VLN* gene family into three groups according to phylogenetic relationships. Further detailed analysis of the soybean VLNs indicated that the ten *GmVLNs* were distributed on 10 of the 20 chromosomes, and their gene structures and protein motifs showed high group specificities. The expression pattern analysis suggested that most *GmVLNs* are widely expressed in various tissues, but three members have a very high level in seeds. Moreover, we observed that the cis−elements enriched in the promoters of *GmVLNs* are mainly related to abiotic stresses, hormone signals, and developmental processes. The largest number of cis−elements were associated with light responses, and two *GmVLNs*, *GmVLN5a*, and *GmVLN5b* were significantly increased under the long light condition. This study not only provides some basic information about the *VLN* gene family but also provides a good reference for further characterizing the diverse functions of *VLN* genes in soybeans.

## 1. Introduction

The actin cytoskeleton is a complex and dynamic network in plant cells that participates in many processes such as vesicle trafficking, cell division and expansion, organelle movement, cytoplasmic streaming, stomata opening, and signal transduction [1,2,3,4,5,6,7,8]. The dynamics and organization of the plant actin cytoskeleton are regulated by different actin binding proteins (ABPs), such as the multifunctional gelsolin/villin/fragmin superfamily, the nucleating proteins Formin and Arp2/3, monomer binding protein profilin, and the severing and depolymerizing proteins ADF and cofilin [5,9,10,11]. These ABPs protein regulate the assembly and disassembly of monomeric actin (G−actin) and filamentous actin (F−actin) at the temporal and spatial levels. Functional analysis of several related ABPs has been reported in *Arabidopsis*. The loss−of−function of *Actin−Depolymerizing Factor 5* (*AtADF5*) decreased the tolerance to drought stress and delayed stomata closure by causing abnormal actin dynamics [12]. Knockdown of *FORMIN3* (*AtFH3*) by RNAi reduced the abundance of actin cables and thus influenced pollen tube growth [13]. Additionally, Fimbrin, as one of the famous members of ABPs, also regulates the actin dynamic. In the *atfim4/atfim5* double mutant, the morphology and length of root hairs were significantly changed [14]. These studies suggested that ABPs play an important role during plant development by regulating actin dynamics.

VLN belongs to the villin/gelsolin/fragmin ABP superfamily and is involved in regulating both the stability and dynamics of actin [15,16,17,18]. The regulatory function of VLN proteins is closely related to their structure. Firstly, VLN proteins have six gelsolin home domains (G1−G6) at the N−terminus and a headpiece domain (VHP) at the C−terminus, both of which contain two conserved actin−binding domains (ABDs). ABD1, spanning the G1 and G2, can bind to two adjacent actin monomers in the filament, while ABD2, located in the VHP, functions to bundle the filaments [5,19,20]. Secondly, Ca^2+^ binding sites are located in all VLN proteins and vary in type and number in different VLNs, but the ability of VLNs to bind to F−actin can be either dependent on or independent of the Ca^2+^/calmodulin. These characteristics permit VLN proteins to regulate the organization and dynamics of actin filaments to form a higher−order structure [9,21,22,23]. In plants, the first functionally characterized VLN homologs were known from the Lily (*Lilium brownie*) and named 115−ABP and 135−ABP, which can bind to the F−actin [24,25,26]. Further studies revealed that 135−ABP plays crucial regulatory roles in the F−actin arrangement of pollen tubes and the cytoplasmic architecture and actin filament organization of root hair [21,25]. In species including *Arabidopsis*, *Rice* (*Oryza sativa*), and *Cotton* (*Gossypium hirsutum*), VLNs were also found to regulate the actin cytoskeletons by nucleating, severing, binding, capping, and depolymerizing [23,27,28]. There are five VLNs in both *Arabidopsis* and *Rice*, and fourteen in *Cotton*; these genes are widely expressed in a variety of tissues [27,29,30,31], but only some of them have been functionally characterized. In *Arabidopsis*, loss−of−function of *VLN1* and *VLN4* caused longer and shorter root hairs, respectively, indicating they regulate the root hair growth in different ways [32]; VLN2 and VLN3 have been reported to regulate the normal development and organ morphogenesis of plants [33,34]; and VLN5 has an important role in the pollen tube growth [35]. In other species, it is known that loss−of−function mutations of the rice *OsVLN2* gene lead to dwarfing and organ curling phenotypes [28], while overexpression of cotton *GhVLN4* in *Arabidopsis* increased the length of cells in the root, root hairs, and pollen tubes [27]. These studies suggest that *VLN* plays an important role in plant development.

To date, there are few reports about VLN proteins in legume species, but some other ABPs and related proteins participating in cytoskeleton dynamics have been reported, such as ADFs, ARPs, and Formin protein [36,37,38]. In *Lotus japonicus*, both Nap1 and Pir1 have been demonstrated to regulate the actin cytoskeleton rearrangement of root. The organization of the actin cytoskeleton was disturbed in *nap1* and *pir1* mutants, causing abnormal rhizobial infection and nitrogen fixation [39]. SCARN, belonging to a novel class of SCAR proteins, plays a critical role in the reorganization of the microfilament cytoskeleton by directly binding and activating the ARP2/3 complex. When the *SCARN* gene is mutated, the formation of infection threads is reduced, affecting the nitrogen fixation ability of root nodules [40]. Additionally, in *M. truncatula*, the actin cytoskeleton coordinates numerous cellular processes in the development of nitrogen−fixing nodules has been reported, and the *SYMBIOTIC FORMIN 1* (*SYFO1*) mutation can abolish the infection chambers and infection threads; silencing the *MtARP2/3* also causes defects in symbiote development and disrupts the nitrogen−fixing units [36,38,41]. Based on these studies, the actin cytoskeleton is necessary for regulating numerous cellular processes during the development of legume plants. VLNs, as a major class of ABP proteins, may also have important roles in these processes in legumes. In soybean, *GmVLN4* was reported to be involved in stress responses, strengthening the resistance to drought, but it is unclear whether *GmVLN4* affects the stability and development of the soybean microfilament cytoskeleton [42]. Soybean is a leading source of feeding, vegetable protein, and oil around the world [43,44,45], and understanding the regulatory process of VLN−related cytoskeleton is important for soybean breeding.

In this study, we systematically investigated the characteristics of GmVLNs by performing a phylogenetic tree, amino acid sequence, three−dimensional prediction, gene structure, and conserved domain analysis. Protein interaction network prediction indicates that GmVLN is involved in cytoskeletal regulation and multiple signal response pathways. Ten *VLN* genes were discovered in the soybean genome, and chromosome localization analysis showed that they were distributed on 10 chromosomes of soybean. The expression pattern analysis suggested that most *GmVLNs* are widely expressed in different tissues, with a higher transcription level in the pod and the axillary bud. It is worth noting that *GmVLN2b*, *GmVLN5a*, and *GmVLN5b* are highly expressed in developing seeds; this clue points out that they possibly take part in seed development regulation. Moreover, the cis−element analysis revealed that the promoters of GmVLNs were enriched with different cis−elements that are associated with plant development, MYB binding, hormones, stress, and light responses. The expression level of *GmVLN5a* and *GmVLN5b* was significantly increased under the long light condition, indicating that these two genes may play an important role in light responses in soybean. In short, this study provided some systematic and basis information for further dissecting the gene function of *GmVLNs* for cytoskeletal dynamics regulation and identifying the promising locus among VLN subgroups for molecular breeding of soybeans in the future.

## 2. Results

### 2.1. Isolation and Characterization of the GmVLNs

**Figure 1:**
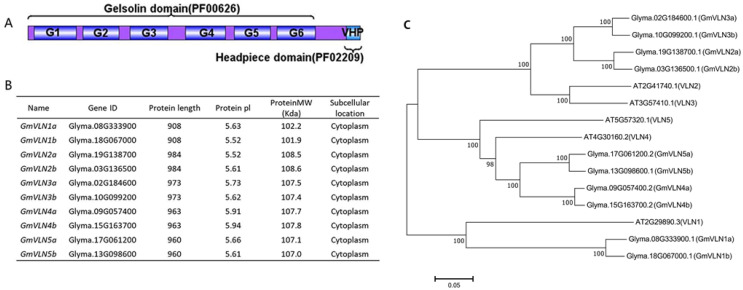
Isolation and characterization of the GmVLNs. (**A**) The VLN harboring the conserved gelsolin domain (PF00626) and headpiece domain (PF02209). (**B**) The physical and chemical properties of GmVLNs. (**C**) Evolutionary relationship between VLN proteins in *Arabidopsis* and *G.max*.

In order to identify the VLN in soybean, the protein sequences corresponding to the gelsolin domain (PFAM family PF00626) and the VHP domain (PFAM family PF02209) profile from Pfam website are used as query sequences, which contain six homology gelsolin domains and a C−terminate headpiece domain (Figure 1A). We used the query sequence to perform the further blast with the soybean protein database by Several Sequences to a Big Database of TBtools, and an NCBI Batch CD−search is used to remove redundant sequences. Finally, the ten soybean VLN were identified. We named soybean VLN proteins as GmVLN1a to GmVLN5b based on their homology with VLN proteins in *Arabidopsis*, and the encoding genes for these GmVLN proteins were given the names *GmVLN1a*, *GmVLN1b*, *GmVLN2a*, *GmVLN2b*, *GmVLN3a*, *GmVLN3b*, *GmVLN4a*, *GmVLN4b*, *GmVLN5a*, and *GmVLN5b*, respectively (Figure 1B,C). 

**Figure 2:**
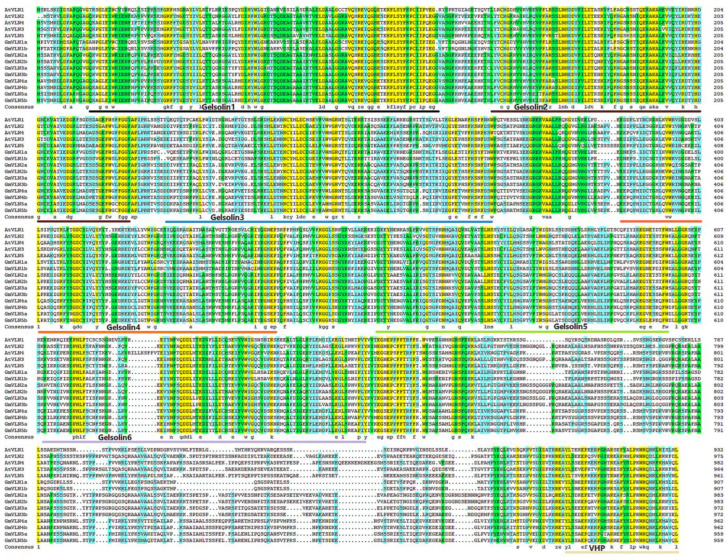
Amino acid alignment of GmVLN proteins. The amino acid alignment of GmVLNs revealed that there are a series of conserved domains. All sequences corresponding to conserved domains are marked with lines of different colors.

In order to better comprehend the GmVLNs, the physical−chemical properties were analyzed, such as the length of the amino acid, the protein isoelectric point (pl), the molecular weight (MV), and the subcellular localization. We found that the protein of GmVLNs varies from 908 to 984 amino acids in length. The estimated molecular mass ranged from 101.9 to 108.6 kDa, and GmVLN2b had the longest length and the highest molecular mass. The predicted isoelectric points of the GmVLNs varied from 5.52 to 5.94; all of them had isoelectric points greater than 5. Furthermore, a preliminary prediction of their subcellular locations revealed that they were all in the cytoplasm. There is an interesting finding: the two paralogous genes of *GmVLNs*, such as *GmVLN1a* and *GmVLN1b*, have the same protein length but differing molecular weights and isoelectric points. (Figure 1B). Moreover, using the VLN protein sequence of *Arabidopsis* as a reference sequence, multiple sequence linearization analysis was performed on soybean, and it was found that all ten GmVLNs possess six typical gelsolin domains and one headpiece domain (Figure 2). The above findings suggest that the similarities and differences in protein structure and characteristics among GmVLNs may confer conservation and diversification of gene function in soybean *GmVLNs*.

### 2.2. Phylogenetic Analysis of GmVLNs and Their Orthologs from Other Representative Plant Species

**Figure 3:**
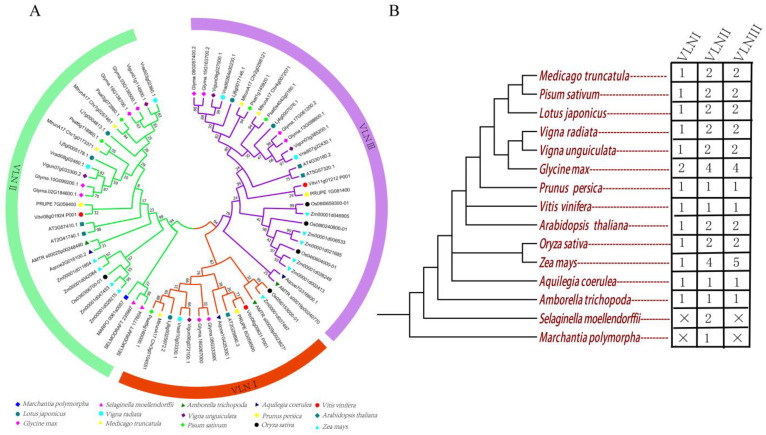
Phylogenetic analysis of GmVLNs and their orthologs from other model plant species. (**A**) Representative species evolution tree. (**B**) The phylogenetic tree−related information for all 70 *VLN* genes of *Medicago truncatula* (*Mt*, 5 members), *Pisum sativum* (*Ps*, 5 members), *Lotus japonicas* (*Lj*, 5 members), *Vigna radiate* (*Vr*, 5 members), *Vigna unguiculata* (*Vu*, 5 members), *Glycine max* (*Gm*, 10 members), *Prunus persica* (*Pp*, 5 members), *Vitis vinifera* (*Vv*, 3 members), *Arabidopsis thaliana* (*At*, 5 members), *Oryza sativa* (*Os*, 5 members), *Zea mays* (*Zm*, 10 members), *Aquilegia coerulea* (*Ac*, 3 members), *Amborella trichopoda* (*Amt*, 3 members), *Selaginella moellendorffii* (*Sm*, 2 members), and *Marchantia polymorpha* (*Mp*, 1 members) by MEGA7.0.

In order to elucidate the evolutionary relationship between the GmVLNs and their orthologs from other representative plant species, we download the protein database of lower plants and higher plants, monocotyledons and dicotyledons, and several widely studied legume species to perform phylogenetic analysis; the detailed information of all these species is listed in supplementary Table S1. To acquire candidate VLN protein sequences, the query sequences were used to blast the protein database of every species with TBtools. Candidate protein sequences contain the conserved gelsolin domain, and the headpiece domain was used to perform phylogenetic analysis. The phylogenetic analysis results showed that 70 VLNs were classified into three VLN subclasses (VLNI to VLNIII), VLNI contained 14 *VLN* genes, both VLNII and VLNIII each contained 28 *VLN* genes, and the number of *VLN* varies among different species (Figure 3A,B). However, we have not screened any ortholog sequence of VLNs in lower plants such as *Porphyra umbilicalis*, *Chlamydomonas reinhardtii*, and *Ostreococcus lucimarinus*, which indicates that the VLN may have been present after plant terrestrialization during the evolution. Among the six legume species, except for soybeans, which contain ten VLNs, other legume species all possess five VLNs. This result echoes the fact that soybeans are ancient tetraploids, while other leguminous crops are diploids. The results of evolutionary analysis indicate that VLN diverged during the evolution of different species, but was very conservative in the evolution of the legume.

### 2.3. Collinearity and Chromosomal Location Analysis of GmVLNs

**Figure 4:**
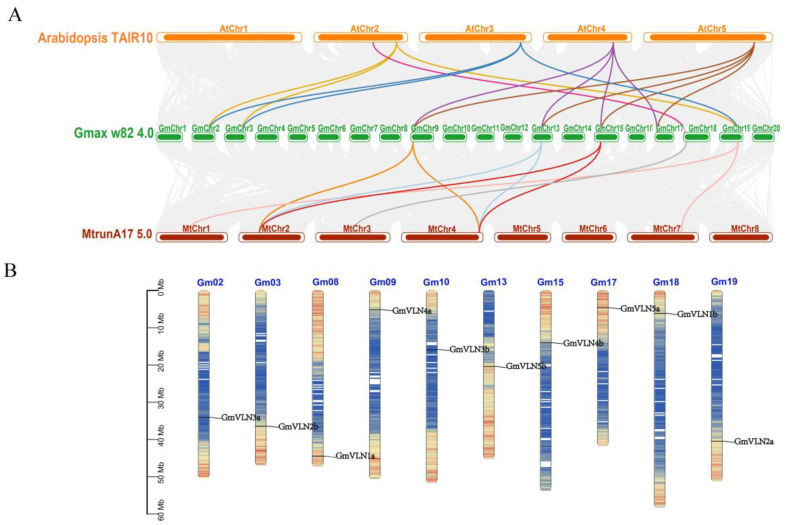
Collinearity and chromosomal location analysis of *GmVLNs*. (**A**) The model plants *A. thaliana* and *M. truncatula* were chosen to produce collinearity with *G. max*; each color line represents one or multiple collinearities. The gray lines in the background indicate the collinear blocks between soybean and other species, and the highlighted lines represent the collinear blocks of *VLN* genes. (**B**) The physical locations of *GmVLNs* on chromosomes. The scale on the left indicated the genomic length in megabases (Mb).

To further explain soybean VLNs underlying function mechanism, the genomes of *A. thaliana*, *M. truncatula*, and *G. max* were used to do the collinearity analysis. There are fifteen orthologous pairs of *VLNs* between the *A. thaliana* and *G. max* genomes. Several *VLN* genes in soybean have synteny with a same *VLN* gene in *A. thaliana*, which implies that these *GmVLNs* may duplicate from the same ancestor during the evolution. Additionally, there are nine orthologous pairs of *VLNs* between the *G. max* and *M. truncatula* genomes. The *VLN* genes of soybean are located on 02 and 03 chromosomes without any collinearity with the *M. truncatula* genome, which suggests that there is no orthologous (Figure 4A). According to these results, it suggests that the VLNs were relatively conserved between *A. thaliana* and *M. truncatula*, and *G. max* during the evolution of higher plants. In the chromosomal location analysis, we found the ten *VLN* genes were unevenly scattered on the 10 of 20 chromosomes in soybean (Figure 4B). In chromosomes 09, 17, 18, 02, 03, 08, and 19, the *VLNs* are located on the terminal of chromosomes. However, on chromosomes 10, 13, and 15 the *VLNs’* locations are near the centromere of the chromosomes.

### 2.4. Protein Interaction Network Construction and Three−Dimentional Prediction

**Figure 5:**
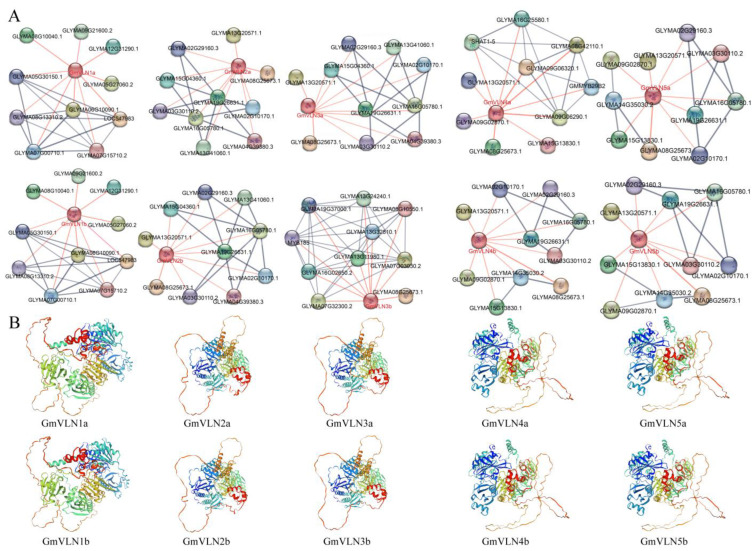
Protein interaction network construct and three−dimensional prediction of GmVLNs proteins. (**A**) Protein−protein interaction network constructed by STRING, where each node represents a protein and each edge represents an interaction, and the red line points to the soybean VLN direct interaction protein. (**B**) Three−dimensional prediction of GmVLNs proteins by the SWISS MODEL.

In order to further understand the role of the GmVLN family in plant development, we conducted protein interaction network prediction and three−dimensional structure prediction for all GmVLN proteins. It was found that most of the interactions with GmVLN proteins are cytoskeletal proteins, such as GLYMA02G29160, GLYMA13G41060, GLYMA15G04360, GLYMA19G26631, and GLYMA04G39380. This result is consistent with the conclusion reported in other studies that VLN regulates cytoskeletal composition by interacting with Actin−related proteins. In addition, GmVLN proteins also interact with development, stress response, hormones, and MYB proteins, indicating that GmVLN is involved in multiple signal response pathways. Detailed interaction protein information is exemplified in the supplementary Tables S6 and S7. In addition to the abovementioned proteins, there are many others that have not been identified for their protein families, including GLYMA05G30150, GLYMA12G31290, SHAT1−5, GLYMA09G06320, GLYMA08G42110, GLYMA13G11980, and GLYMA08G10550, preliminary functional predictions indicate that these proteins also participate in important physiological processes within the cell (Figure 5A). 

In the 3D structure prediction analysis, three parameters, GMQE, coverage, and seq−identity, are combined for template screening. The larger these parameters of the template, the more accurate the prediction will be, and the selection process will select the one with the highest score. In our study, all GMQE values were greater than 0.75, ranging from 0.77 to 0.81; the coverage rate is 100%; and the seq−identity is between 81.77% and 100%, all of which have high reliability. In the same subgroup, the prediction templates used are the same, consistent with their evolutionary conservatism mentioned above, indicating that the prediction template we have chosen is reliable. The results indicate that GmVLN proteins in the same subgroup are highly similar in spatial structure, such as GmVLN1a and GmVLN1b, GmVLN2a to GmVLN3b, and GmVLN4a to GmVLN5b. There are significant differences in the three−dimensional structure of different subgroups. These structural similarities and differences may be related to their functions (Figure 5B), and the parameters and usage models for predicting the three−dimensional structure of proteins are presented in supplementary Table S8.

### 2.5. Gene Structure Analysis of GmVLNs and the Conserved Motif and Domain Analysis of GmVLNs

**Figure 6:**
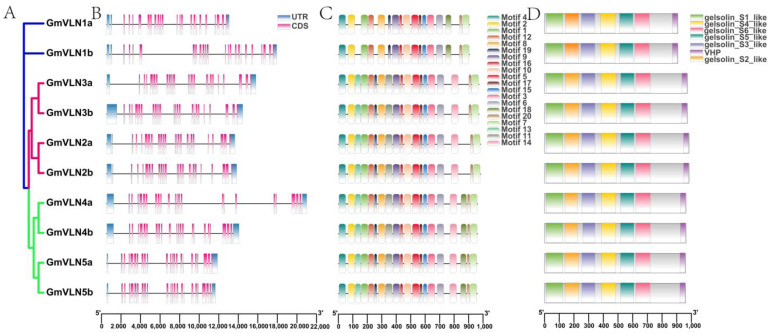
Gene structure of *GmVLNs*, motif, and conservative domain analysis of GmVLNs. (**A**) Intraspecific evolutionary tree of soybean. (**B**) The gene structure of *GmVLNs*, the pink boxes, blue boxes, and black lines represent the exon, UTR, and intron, respectively. (**C**) The motif composition of GmVLNs, with conserved motifs in the soybean VLN proteins indicated by colored boxes. (**D**) Conserved domain of the GmVLNs, with colored boxes representing the conserved protein domain.

To further gain insight into the structural properties of the *GmVLNs* and their encoding proteins, we performed the gene structure, conserved motif, and domain analysis of GmVLNs (Figure 6). We found that the pattern of gene structure, conserved motif, and domain was remarkably similar among members of the same group, but only slightly different between groups. The gene of *GmVLNs* of VLNI and VLNIII contains 22 exons and 21 introns, and VLNII contain 23 exons and 22 introns (Figure 6B). This result is consistent with the evolutionary result of the GmVLN, where VLNI and VLNIII are on the same branch, while VLNII are alone in the same branch. Moreover, the lengths of the GmVLN sequences, exons, and introns are different. In general, in the same group with similar structural features, it indicates that gene function and the phylogenetic relationship among the *GmVLN* genes are highly conserved.

The motif and domain of the GmVLN proteins were also studied to better understand their conservation and diversification. A total of 20 conserved motifs were named Motifs 1−20. We found that in the same group, the type and number of GmVLN are highly similar, and different subgroups of the motif number of GmVLN vary from 17 to 20 (Figure 6C). GmVLN1a and GmVLN1b have 17 motifs, GmVLN2a to GmVLN3b have 19 motifs, and GmVLN4a to GmVLN5b have 20 motifs. Furthermore, we discovered that the conserved domain of GmVLN is highly conserved, with all of them having six geisolin domains and a headpiece domain (VHP), and the VHP location is similar in the same group (Figure 6D). All of these structural analyses indicate that GmVLNs and their encoding proteins have synergistic structures, and the difference in these structures may explain the function difference. This will provide the structural basis for their gene function conservation.

### 2.6. Expression Patterns of GmVLNs in Different Organs in Soybean

**Figure 7:**
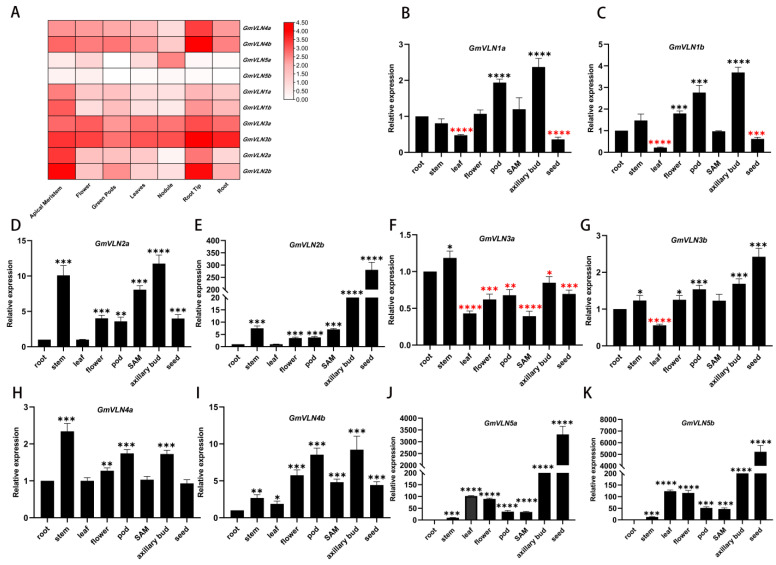
Expression patterns of *GmVLNs* in different tissues and organs of soybean. (**A**) The heat−map of transcriptional level of *GmVLNs*, the data come from the microarray, and the heat map was constructed with TBtools. (**B**–**K**) The tissues expressional pattern of all *GmVLNs* was detected by RT−qPCR. Error bar means ± SD, Asterisks indicate a significant difference between root−other tissues (*t*−test, 0.01 < * *p* < 0.05, 0.001 < ** *p* < 0.01, 0.0001 < *** *p* < 0.001, **** *p* < 0.0001, red asterisks represent a significant decrease, while black asterisks represent a significant increase).

To principally investigate the probable gene function of GmVLNs in various soybean tissues, the tissue−specific expression data of GmVLNs were first downloaded from the Legume Information System database, which includes the transcriptional profiles of the shoot apical meristem, flower, green pods, leaves, nodule, root tip, and root (Figure 7A). This result revealed that most of the GmVLNs were constructively expressed in all tissues, and the paralogous genes showed similar expression patterns, such as GmVLN1a and GmVLN1b, GmVLN2a and GmVLN2b, GmVLN3a and GmVLN3b, GmVLN4a and GmVLN4b, and GmVLN5a and GmVLN5b, and there are only small differences in expression between them. Both GmVLN3a and GmVLN3b had high expression levels in various tissues, too, especially in the shoot apical meristem and root tip. Subsequently, RT−qPCR was performed to further analyze the expression pattern of these genes in different organs of soybean (Figure 7B–K). The results are consistent with the above results, and paralogous gene expression patterns are similar. A special discovery we found was that GmVLN2b (Figure 7E), GmVLN5a (Figure 7J), and GmVLN5b (Figure 7K) were highly expressed in the seed, which indicated that these genes may play an important function during seed development. These results indicate that the GmVLN function in the same branch may be very conservative, and different branches may have different functions.

### 2.7. Promoter Cis−Element Analysis and Expression Pattern of GmVLNs under the Different Lighting Periods

**Figure 8:**
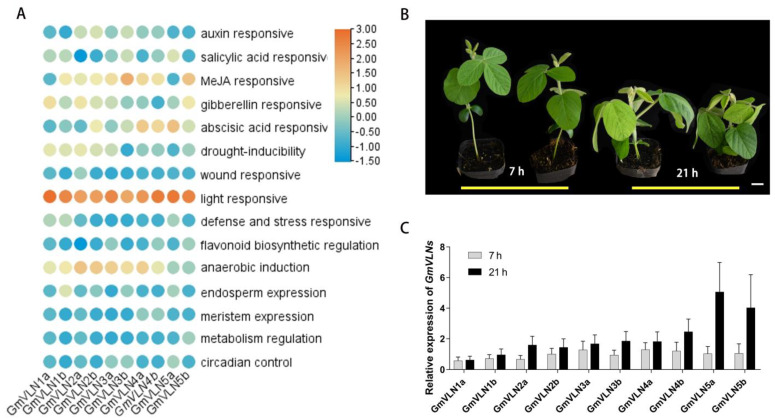
Promoter cis−element analysis and expression pattern of *GmVLNs* under the different lighting periods. (**A**) The 3.5 kb upstream region of the start code was used to do the cis−element analysis with Plant−CARE. Hormone−responsive elements, stress−responsive elements, light−responsive elements, and other related elements are present in different colors. (**B**) The one−month−old seedlings of soybean, which are under 7 or 21 h light growth conditions. (**C**) The transcriptional level of *GmVLN* genes under different light period conditions.

In order to further investigate the possible biological function of GmVLNs, we screened and analyzed the cis−elements in the predicted promoter region of the GmVLNs. The 3.5 kb sequence upstream of the start code of the GmVLNs was used as the promoter region for the analysis. The cis−elements screening of GmVLNs was performed via the Plant−CARE online website, and the heat map was constructed by TBtools (Figure 8A). We found that these identified cis−elements in the promoter region of GmVLNs are related to biological processes such as auxin, salicylic acid, JA, gibberellin, abscisic acid responses, MYB binding, light, stress, wound, and other responses. Among these cis-elements, the light response elements are the most pronounced. Elements of the TGA−element, TCA−element, CGTCA−motif, GARE−motif, ABRE, TATC−box, TGACG−motif, P−box, AE−box, chs−CMA2a, and TC−rich repeats were predicted to be involved in the hormone signaling pathways. MYB binding site elements include MBS, MBSI, CCAAT−box, and MRE. The stress response (here, stress pointed out the light, drought, low temperature, defense, and stress anaerobic induction) elements include LTR, ATC−motif, TCT−motif, Sp1, G−box, CAG−motif, GATA−motif, Box4, AE−box, TCCC−motif, AT1−motif, TC−rich repeats, GT1−motif, chs−CMA1a, GA−motif, ACE, LAMP, Gap−box, and GTGGC−motif. In addition to the abovementioned cis−elements, it also contains the cis−elements related to endosperm expression, meristem expression, metabolism regulation, and circadian control elements. Cis−element analysis showed that the GmVLNs may involve various signal pathways and biological processes. Until now, there is no clear evidence to verify these speculations, so it will be promising to analyze the gene function of *GmVLNs*, which is related to external and internal environment signal responses.

To identify whether light responsive elements are important for *GmVLN* gene expression, seedlings were grown under different light length conditions. We found that the plants under short light conditions were taller and more slender compared with the plants growing under long light conditions, which were shorter and more robust (Figure 8B). The results showed that the seedling architecture may be associated with a light response element (Figure 8B). Additionly, RT−qPCR was performed to detect the *GmVLNs* gene expression level. The results showed that the transcriptional level of *GmVLN5a* and *GmVLN5b* was significantly higher for the seedlings that were grown under long light period conditions when compared to the seedlings that were grown under short light period conditions (Figure 8C). These results indicated that the screened light response−related cis−elements on the promoters of *GmVLN5a* and *GmVLN5b* may really be responsible for the transcriptional differences, and the *GmVLN5a* and *GmVLN5b* genes may play an important role in the light response of soybean. Further analysis was required to further uncover the gene function of these promising genes in the future.

## 3. Discussion

The *VLN* gene family is an important family possessing actin−binding domain and involved in different aspects of plant growth and development. It is a group of regulators that mediate actin dynamics by polymerizing and depolymerizing actin filament [46]. Under different states of development and environmental stress, VLN can rearrange filament via severing and bundling actin filament [6,33,34,47]. However, before our study, there had been no genome−wide, in−depth study of the VLN gene family reported in the soybean, which is an important and widespread legume crop. In this study, we showed that 10 GmVLNs of soybean were grouped into three subgroups and their gene structures and protein motifs showed high group specificities. The genome-wide identification and characterization of the soybean *VLN* gene family is an essential step for further exploring the function of this gene family in depth.

There are some studies about the roles of *VLN* genes in regulating plant architecture, which is related to the final yield of crops. In *Oryza sativa*, OsVLN2 participates in regulating plant height and grain plumpness. Loss−of−function of *OsVLN2* led to a semi−dwarfed phenotype, which was caused by the shorter first and second internodes when compared with WT. In addition, the *osvln2* mutant also showed twisted leaf sheaths and caved grains, which resulted in a decreased seed setting rate and 1000−grain weight [28]. All VLN genes have been reported to regulate the different tissues development by controlling the organization of actin filaments in *A. thaliana* [6,18,33,34,35]. *AtVLN2* and *AtVLN3* are functional redundancies to regulate the plant architecture. The *vln2/vln3* double mutant produces a twisted phenotype in root, stem, leaf, pod, and inflorescences [33,34]. For crops, the twisted organs may be detrimental for plant photosynthesis, biomass, and harvest. In the intercropping system between maize and soybean, proper plant type is important for increasing soybean yield [48]. Furthermore, AtVLN5 was reported to regulate pollen germination and pollen tube growth during the reproductive stage [35]. According to the above studies, it appeared that VLNs play a prominent role in crop yield regulation, which will help us decipher the genetic basis for the improvement of crops. In this study, the phylogenetic analysis showed that all VLNs are clustered into three subgroups. In subgroup I, *Arabidopsis* and all diploid legumes contain one member, while the ancient tetraploid soybean contains two members; in subgroup II and III, *Arabidopsis* and all diploid legumes contain two members, and the soybean has four members. This indicates that the gene number of *VLNs* is rather constant in legumes and *Arabidopsis*, suggesting that there may be some conservation of function in this gene family. Our research shows that the 10 *GmVLNs* of soybean are widely expressed in different tissues and organs, indicating important roles in regulating various aspects of soybean growth and development. It is worth noting that the three genes *GmVLN2b*, *GmVLN5a*, and *GmVLN5b* are highly expressed in seeds, and they may be involved in the regulation of seed traits, which are potential factors for soybean yield regulation. To cultivate high−quality and high-yield soybean breeds, we need to do more exploration to uncover the function of the *VLN* gene in plant architecture and crop yield in the future.

In addition, strengthening the stress response is considered an important way for improving crop agronomic traits and increasing yield, such as resistance to high temperatures, cold, salt, drought, and the hormone response. The technologies of quantitative trait loci (QTL), mapping, CRISPR, and high−throughput were used to research the gene of soybean stress response to cultivate high−quality soybeans [49,50]. Many members of the *VLN* gene family have been shown to be involved in various stress responses. Loss−of−function of *VLN1* in *Arabidopsis* showed better growth compared with Col−0 under the osmotic stress. However, ectopic overexpression of *GhVLN4* in *Arabidopsis* exhibited higher resistance to salt and drought than the wild−type plants [27]. In our study, the cis−element analysis suggested that GmVLNs may be involved in hormone, light, drought, and defense responses (Figure 8). To examine this hypothesis, this study conducted an analysis of the impact of light stress on soybean growth and development. It was found that light treatment induced dwarfing, accompanied by significant changes in the expression of *GmVLN5a* and *GmVLN5b*. Given that both of these genes and the cotton GhVLN4 belong to the subgroup III, it is reasonable to believe that these genes are evolutionarily homologous and may be involved not only in light stress responses but also in other stress responses. Although other *VLN* genes show no differences in expression levels in response to light stress, they may be necessary for other stress responses or developmental processes, which needs to be further tested in future research.

## 4. Materials and Methods

### 4.1. Identification and Data Collection of Soybean VLN Family

To identify all the possible VLN in soybean, we downloaded soybean protein data from the Phytozome website (Phytozome info: G.max Wm82.a4.v1 (doe.gov) accessed on 8 July 2022) [51]. Firstly, searching for PF00626 or PF02209 on the Pfam website resulted in a large number of domain organization models. A model consisting of 6 gelsolins and 1 VHP was selected, with a UniProt of A0A7R5KBD8 and a protein sequence downloaded as a reference sequence (Pfam is now hosted by InterPro (xfam.org) accessed on 25 July 2022) [52]. According to the reference sequence to identify all members of the soybean VLN in Several Sequences to a Big File of TBtools, the number of threads was set to 2, the E−value was set to 1 × 10^−5^, the number of hits was set to 500, and the number of alignments was set to 250, and a total of 12 protein sequence IDs have been extracted that may be highly homologous to the reference sequence. Protein sequences of 12 protein IDs were then extracted with Fasta Extract of TBtools. To strengthen the data reliability, all conserved domains of soybean VLN were predicted by the NCBI batch CD search (Welcome to NCBI Batch CD-search nih.gov) [53], the expected value threshold was set at 0.01, and the sequence that contained the gelsolin domain and the headpiece domain was selected and the other was deleted. These selected sequences were conformed again through the SMART website (SMART: Main page (embl-heidelberg.de) accessed on 5 August 2022) [54].

### 4.2. Basic Physicochemical Properties, Amino Acid Alignment Analysis, and 3D Structure Prediction of Soybean VLN 

To further understand the characteristics of soybean VLN, the physical and chemical parameters of soybean VLN protein were calculated by the online software ExPASY (ExPASy-ProtParam tool) [55], including molecular weight (MW), isoelectric point (Pl), and amino acid numbers. The amino acid alignment and sub−location were also analyzed in DNAMAN Version 10 and Plant-mPLoc server (sjtu.edu.cn (accessed on 10 August 2022)) [56], and this was performed separately. The three−dimensional structure of GmVLNs proteins was predicted using the online tool ExPaSy SWISS−MODEL (SWISS-MODEL Interactive Workspace (expasy.org) accessed on 25 March 2023).

When predicting the three−dimensional structure of proteins, global model quality evaluation (GMQE), coverage, and sequence identity (Seq Identity) are important screening parameters. The GMQE is between 0 and 1, and the closer it is to 1, the better the quality of the model used. The coverage rate represents the degree to which the target protein sequence is covered by the template protein sequence; the seq identity represents the alignment and matching degree of amino acids; and the higher the identity, the more reliable the model, and the more accurate the predicted structure.

### 4.3. Phylogenetic, Collinearity, Chromosomal Location Analysis of GmVLNs Gene

We downloaded 18 species’ protein data from different websites to understand the evolutionary relationship among representative species, screening candidate protein sequences from different species based on the methods and parameters in Section 4.1. The sequence was selected that contained the conserved gelsolin domain and headpiece domain when using NCBI batch CD−search and the SMART website to predict. In this study, the phylogenetic tree was constructed with the maximum likelihood (ML) method in MEGA. A phylogenetic tree visualized result was shown on MEGA7.0. We also downloaded the whole genome data of *G. max*, *Arabidopsis thaliana*, and *M. truncatula*, and genome sequences were extracted according to transcript IDs by using the Fasta Extract of TBtools. The collinearity relationship between two species is established with a One−Step MCScanx of TBtools: the CPU for BlastP was set to 2, the E−value was set to 1 × 10^−10^, and the number of blast hits was set to 5. We then merged the links, gff, and multiple synteny files using Text Merge for MCScanx of TBtools. Finally, the triple species collinearity relationship was constructed with Multiple Synteny Plot [57]. In addition, the chromosomal location analysis of the GmVLNs gene was also complete through Gene Location Visualize from GTF/GFF of TBtools.

### 4.4. Gene Structure, Conserved Motif and Conserved Domain Analysis

The soybean VLN family protein gene sequence was downloaded from the Phytozome websites (Phytozome info: G. max Wm82. a4. v1 (doe.gov) accessed on 8 July 2022) [51] and extracted using Fasta Extract of TBtools, the gene sequence was used to construct the gene structure, and the protein sequence was used for motif and domain analysis. Based on the online website NCBI batch, CD search prediction conserved domain (Welcome to NCBI Batch CD-search (nih.gov) accessed on 28 July 2022); MEME (MEME-Submission form (meme-suite.org) accessed on 28 July 2022) [58] prediction conserved motif; and the evolutionary tree within the species were established by One Step Build a ML Tree of TBtools using protein sequences. Finally, all these results were visualized in the Gene Structure View of TBtools. 

### 4.5. Protein Interaction Network Diagram Construction and Cis-Element Analysis

To understand plant VLNs function, we based model plants on the soybean protein database and constructed protein interaction networks using an online tool (STRING: functional protein association networks (string-db.org) accessed on 9 May 2023) (confidence limit is 0.4), and all scores for protein interactions were listed in supplementary Tables S6 and S7. The soybean VLNs cis−element prediction was by Plant CARE (Search for cis−acting regulatory elements (ugent.be) accessed on 28 July 2022), and selected a 3.5 kb sequence upstream of the ATG, which was analyzed, and the result was visualized via HeatMap user interface of TBtools.

### 4.6. Different Lighting Period Treatment and Expression Pattern Analysis in Different Tissues

For quantitative PCR expression analysis, the cultivar Williams 82 was used in this study. Soybean seeds were soil−grown in greenhouses under the following controlled conditions: 24 °C day/20 °C night temperature; growth in 21 h light/3 h dark or 7 h light/17 h dark photoperiod 30% to 50% relative humidity; and 150 μE/m^2^/s light intensity. The seedlings were randomly divided into two groups for different lighting periods of 30 days. The shoots of treated samples were collected 30 days after the beginning of the treatment and processed for analysis of the expression patterns of different *VLN* genes. Each tray was replicated three times. Seedlings were grown under the same temperature and humidity, and normal photoperiod (14 h light/10 h dark) conditions were used for tissue specific expression analysis. Tissues of the root, stem, leaf, flower, pod, SAM, seed, and axillary bud were collected and used for relative quantitative analysis.

### 4.7. RNA Extraction and qRT PCR Detection

Sheng Gong’s UNIQ−10 column: Trizol total RNA extraction kit was used to extract RNA, using Nano−Drop 2000 UV spectrophotometer to detect RNA extract quality; Maibo cDNA synthesis kit was used to reverse transcription RNA to obtain cDNA; and the amount of reverse transcription RNA was 2 micrograms, diluted 5× as the qRT−PCR reaction template. qRT−PCR primer design on the NCBI primer blast web page and the primers used in the experiment are shown in Supplementary Table S3. Maibo fast qPCR Master Mix kit was used, where the reaction system is 10 μL and the PCR reaction program has a 95 °C pre−denaturation for 10 min, and then 40 cycles including 95 °C denaturation for 15 s and 60 °C annealing for 1 min were performed. The instrument used was Applied Biosystems 7500, and the experimental results were processed by the 2^−ΔΔCt^ method [59], and every experiment was repeated three times in an independent sample. Significant differences in mean values at different sampling times were determined by Tukey’s pairwise comparison tests, as indicated by different letters in the figures. The graphical representation of the experimental findings was produced using Graphpad.

During RNA extraction, the tissues of roots, stems, leaves, and SAM were all taken from normally growing seedlings at 30 days old, axillary bud tissues from plants at 45 days old, flowers from plants at 60 days old, fruit pods from plants at 70 days old, and seeds from plants at 85 days old. In the qRT experiment, the reference gene *ACT11*(*Glyma.18g290800*) was used.

## 5. Conclusions

In this study, the phylogeny and characteristics of the *VLN* gene in soybean were explored from different aspects. All 10 *GmVLN genes* were divided into three groups. The genes in the same group shared similar evolutionary characteristics, such as three−dimensional structure, gene structure, motifs, and conserved domains, which implies that they may function similarly. Additionally, the *GmVLN* gene was evenly distributed on 10 chromosomes in the soybean genome, and the amino acid alignment is highly similar. Collinearity analysis indicates that the *GmVLNs* gene may have similar functions to the *VLN* genes in model species *Arabidopsis* and *Medicago*. Analysis of expression patterns in different tissues shows that *GmVLN* is widely expressed in different tissues, and different GmVLN members may have functional overlap. The protein interaction network and cis−element analysis shows that GmVLNs may involve different physiological processes, including hormones, stress, and development signal responses. These findings from our work will serve as a solid foundation for future function studies of the *VLNs* gene in soybeans or other species of legumes.

## Data Availability

Not applicable.

## Supplementary Materials

The following supporting information can be downloaded at: https://www.mdpi.com/article/10.3390/plants12112101/s1, Table S2: Characteristics analysis of the 20 conserved motifs in GmVLNs; Table S4: The relative expression of the *GmVLNs* gene in different lighting time treatments: Table S5: The relative expression level of the *GmVLNs* gene in different tissues; Table S9: The raw transcription data of tissues of *GmVLN* Download in LIS (Legume Information System); Table S10: In q−RT detection, the Ct value of soybean *VLN* gene in various tissue.
