# Supplementary material for: Genome-Wide Identification and Expression Analysis of the VILLIN Gene Family in Soybean
Source: Plants (Basel). 2023 May 25;12(11):2101. doi: 10.3390/plants12112101 (PMC10255930; doi:10.3390/plants12112101)
Supplement: Supplementary file 1 [file plants-12-02101-s001.zip › plants-2338659-supplementary.pdf]

Supplementary Table S1. Representative species-related information.

Speices	villin family members	database
Porphyra umbilicalis v1.5	0	Phytozome
Chlamydomonas reinhardtii	0	Ensembl Plants
Ostreococcus lucimarinus	0	Ensembl Plants
Marchantia polymorpha	1	Ensembl Plants
Selaginella moellendorffii	2	Ensembl Plants
Amborella trichopoda	3	Ensembl Plants
Aquilegia coerulea	3	Phytozome
Vitis vinifera	3	Ensembl Plants
Lotus japonicus	5	Ensembl Plants
Vigna radiata	5	Phytozome
Vigna unguiculata	5	Ensembl Plants
Prunus persica	3	Ensembl Plants
Arabidopsis thaliana	5	Phytozome
Glycine max	10	Phytozome
Medicago truncatula	5	MtrunA17r5.0-ANR
Pisum sativum	5	Ensembl Plants
Oryza sativa	5	Ensembl Plants
Zea mays	10	Phytozome

Supplementary Table S2. Characteristics analysis of the 20 conserved motifs in GmVLNs.

name	sequence	length	E-value	position prediction
motif1	RSSLNHDDVFILDTZNKIFQFNGANSSIQERAKALEVVQYJKEKYHEGKC	50	1.9E-319	Gelsolin domain 2
motif2	WJGKDTSQDEAGAAAIKTVELDAALGGRAVQYREIQGHESEKFLSYFKPC	50	2.90E-301	Gelsolin domain 1
motif3	DPHLFSLSFSKGNLKVEEVYNFSQDDLLTEDILILDCHSEIFVWVGQQVD	50	2.10E-279	Gelsolin domain 6
motif4	TKDLDPAFQGAGQKAGLEIWRIENFQPVPVPKSSYGKFYTGDSYIILKTT	50	4.70E-271	Gelsolin domain 1
motif5	TYNEEGVALFRIQGTSPDNMQAIQVDPVASSLNSTYCYILQSGPTVFTWS	50	2.10E-263	Gelsolin domain 5
motif6	KFJEHDALLEGLSPHVPJYIVTEGNEPPFFTRFFSWDHAKAMVLGNSFQR	50	4.60E-257	Gelsolin domain 6
motif7	GDPVPGIDLTKREAYLSSEEFQEKFGMAKEAFYKLPRWKQBKLKMAVDLF	50	1.20E-255	Villin headpiece domain
motif8	LETNKCYJLDCGAEVFVWMGRNTSLEERKSASQAAEEFVAGQGRPKSTRI	50	2.70E-244	Gelsolin domain 3
motif9	PVEEEPPPHJDCTGKJZVWRVNGQEKTLLPAEDISKFYSGDCYIVQYTYP	50	1.10E-238	Gelsolin domain 4
motif10	LASTMVESLKGRPVQARIYEGKEPPQFHAILQPMIVLKGGLSSGYKKYIA	50	1.80E-230	Gelsolin domain 4
motif11	RIIZGYETVSFKSKFDSWPSGSAATVAEEGRGKVAALLKRQGMGVKGLTK	50	3.10E-192	Gelsolin domain 5
motif12	EVAIVEDGKLMADSDSGEFWGLFGGFAPJPRKTIS	41	4.90E-176	Gelsolin domain 2
motif13	IIPLEGGVASGFKKPEEEEFETRLYVCRGKRVVRVKZVPFA	41	1.80E-170	Gelsolin domain 6
motif14	RDMSVGPDRGGPRGRAPALAALAAAFESSNGTKLSTPPPSGRGLGPKRAA	50	9.70E-145	No putative conserved domain
motif15	KPAKEGTESEQFWDALGGKQEYPSKKITR	29	3.00E-101	Gelsolin domain 5

motif16	GEDKEDYLJGTWFGKBSVEED	21	1.60E-71	Gelsolin domain 4
motif17	GNLCSAEDQZLVERMLDLJKP	21	3.30E-57	Gelsolin domain 5
motif18	REPLIPRSQKASPVTPKSNPETSDKEGSVSSRIESLTIQED	41	6.00E-43	No putative conserved domain
motif19	ETIPPKLYSIEKGEAEPVEGE	21	5.60E-42	Gelsolin domain 3
motif20	DEEEDNEGLAIYPYERLKTKS	21	3.60E-41	No putative conserved domain

Supplementary Table S3. The qRT-PCR primer of *GmVLN*.

Primer name	Forward/reverse	DNA sequence(5′→3′)
GmVLN1a	F	CAAACTGCTGGAGCAAACCC
	R	TGTCATACTGAGGAGGGCCA
GmVLN1b	F	TGGCCCTCCTCAGTATGACA
	R	ACTTCTGCGATTCTTGGCCT
GmVLN2a	F	GCATTCCAGGGAGTTGGTCA
	R	AAGCACCTCCTTTGCCTTGT
GmVLN2b	F	AGGTTCCTTTTGCACGGTCT
	R	GCCTTGGCTCTTTCCTGGAT
GmVLN3a	F	GTCAAGCCTGTGGAAGGTGA
	R	GCAACAAACTCCTCAACGGC
GmVLN3b	F	GCATTCCAGGGAGTTGGTCA
	R	TGCCTCCTTTGCCTTGAGTT
GmVLN4a	F	GCTGCAATGCTGGGAAACTC
	R	AGACATGCTGCGAGAGGAAC
GmVLN4b	F	GGATCCAGCTTTCCAGGGAG
	R	AATGGATGTCATGGCGCAGA
GmVLN5a	F	GCCGGATTGGAAATATGGCG
	R	AGCACCAGCTTCATCCTGAC
GmVLN5b	F	ATGCAGCTTTAGGAGGACGG
	R	AGCCCCACCTTCTTGAGGTA
ACT11	F	CGGTGGTTCTATCTTGGCATC
	R	GTCTTTCGCTTCAATAACCCTA

Supplementary Table S4. The relative expression of the *GmVLNs* gene in different lighting time treatments.

Gene ID	Expression level of Lighting 7h	Expression level of Lighting 21h
GmVLN1a	0.5816	0.6305
GmVLN1b	0.7227	0.9588
GmVLN2a	0.6785	1.595

GmVLN2b	1.002	1.445
GmVLN3a	1.274	1.678
GmVLN3b	0.9407	1.858
GmVLN4a	1.291	1.82
GmVLN4b	1.205	2.461
GmVLN5a	1.032	5.065
GmVLN5b	1.047	4.033

Supplementary Table S5. The relative expression level of the GmVLNs gene in different tissues.

Gene ID	root	stem	leaf	flower	pod	seed	Axillary bud	SAM
GmVLN1a	1.23599555	1.801893542	1.009042107	2.292032324	2.292032324	0.777285228	4.618500115	2.598006934
GmVLN1b	2.635237444	4.08410866	1.020285276	5.093981612	7.033282117	1.671556263	9.957339529	1.671556263
GmVLN2a	0.595176697	5.576134777	1.013730222	2.646420918	2.018824698	2.58117748	6.894693307	5.201506449
GmVLN2b	0.575021457	3.674517537	1.025594471	2.171814806	2.681385759	162.9812644	18.6925516	4.240826752
GmVLN3a	1.349918201	1.675662187	1.014025859	0.920477779	0.879214406	0.94430022	1.130355401	0.610218664
GmVLN3b	1.171722715	1.55394983	1.01642622	1.713999767	1.756973318	2.999626991	1.95339025	1.414577816
GmVLN4a	0.621945572	1.521998885	1.015001468	0.842609193	1.084265944	0.62664469	1.18188255	0.722932332
GmVLN4b	0.30090806	0.860676008	1.027280752	1.892686624	2.435501139	1.407582581	2.654769628	1.623865923
GmVLN5a	0.006076708	0.061545663	1.012658527	0.522462625	0.197004916	19.09358694	2.356563108	0.211363837
GmVLN5b	0.005666301	0.064885746	1.009042107	0.583047331	0.229410885	27.11691516	3.059741466	0.262549885

Supplementary Table S6. Predicted Soybean VLN interaction proteins and their scores.

VLN protein	Interaction protein	Score	VLN protein	Interaction protein	Score
GmVLN1a	GLYMA09G21600.2	0.612	GmVLN1b	GLYMA09G21600.2	0.612
	GLYMA08G10040.1	0.612		GLYMA08G10040.1	0.612
	GLYMA07G00710.1	0.604		GLYMA05G30150.1	0.548
	GLYMA05G30150.1	0.548		GLYMA08G13310.2	0.548
	GLYMA08G13310.2	0.548		GLYMA07G00710.1	0.604
	GLYMA06G10090.1	0.628		GLYMA06G10090.1	0.628
	GLYMA07G15710.2	0.546		GLYMA07G15710.2	0.546
	LOC547983(GLYMA04G10090.1)	0.628		LOC547983(GLYMA04G10090.1)	0.628
	GLYMA05G27060.2	0.612		GLYMA05G27060.2	0.612
	GLYMA12G31290.1	0.612		GLYMA12G31290.1	0.612
	GLYMA09G21600.2	0.612		GLYMA09G21600.2	0.612

	GLYMA08G10040.1	0.612		GLYMA08G10040.1	0.612
	GLYMA07G00710.1	0.604		GLYMA05G30150.1	0.548
	GLYMA05G30150.1	0.548		GLYMA08G13310.2	0.548
	GLYMA13G20571.1	0.555		GLYMA13G20571.1	0.555
	GLYMA02G29160.3	0.512		GLYMA08G25673.1	0.603
	GLYMA15G04360.1	0.53		GLYMA03G30110.2	0.512
	GLYMA03G30110.2	0.512		GLYMA04G39380.3	0.512
	GLYMA19G26631.1	0.534		GLYMA02G10170.1	0.512
	GLYMA16G05780.1	0.534		GLYMA16G05780.1	0.534
GmVLN2a	GLYMA13G41060.1	0.53	GmVLN2b	GLYMA19G26631.1	0.534
	GLYMA04G39380.3	0.512		GLYMA13G41060.1	0.53
	GLYMA02G10170.1	0.512		GLYMA02G29160.3	0.512
	GLYMA08G25673.1	0.603		GLYMA15G04360.1	0.53
	GLYMA13G20571.1	0.555		GLYMA13G20571.1	0.555
	GLYMA02G29160.3	0.512		GLYMA08G25673.1	0.603
	GLYMA13G20571.1	0.555		GLYMA07G32300.2	0.594
	GLYMA08G25673.1	0.603		GLYMA16G02650.2	0.594
	GLYMA03G30110.2	0.512		MYB185(GLYMA19G44660.1)	0.593
	GLYMA04G39380.3	0.512		GLYMA19G37000.1	0.593
	GLYMA02G10170.1	0.512		GLYMA13G11980.1	0.594
GmVLN3a	GLYMA16G05780.1	0.534	GmVLN3b	GLYMA13G32810.1	0.593
	GLYMA19G26631.1	0.534		GLYMA13G24240.1	0.594
	GLYMA13G41060.1	0.53		GLYMA08G10550.1	0.592
	GLYMA02G29160.3	0.512		GLYMA07G03030.2	0.594
	GLYMA15G04360.1	0.53		GLYMA08G25673.1	0.603
	GLYMA13G20571.1	0.555		GLYMA07G32300.2	0.594
	GLYMA13G20571.1	0.555		GLYMA13G20571.1	0.555
	GLYMA09G02870.1	0.548		GLYMA09G02870.1	0.548
	GLYMA08G25673.1	0.691		GLYMA15G13830.1	0.548
	GLYMA15G13830.1	0.548		?GLYMA14G35030.2	0.513
	GMMYB29B2(GLYMA20G35180.1)	0.595		?GLYMA08G25673.1	0.691
GmVLN4a	GLYMA09G06290.1	0.728	GmVLN4b	GLYMA03G30110.2	0.512
	GLYMA08G42110.1	0.728		GLYMA19G26631.1	0.534
	GLYMA09G06320.1	0.731		GLYMA16G05780.1	0.534
	GLYMA16G25580.1	0.599		GLYMA02G29160.3	0.512
	SHAT1-5(GLYMA16G02200.2)	0.599		GLYMA02G10170.1	0.512
	GLYMA13G20571.1	0.555		GLYMA13G20571.1	0.555
	GLYMA13G20571.1	0.555		GLYMA13G20571.1	0.555
	GLYMA09G02870.1	0.548		GLYMA15G13830.1	0.548
GmVLN5a	GLYMA14G35030.2	0.513	GmVLN5b	GLYMA09G02870.1	0.548
	GLYMA15G13830.1	0.548		GLYMA14G35030.2	0.513
	GLYMA08G25673.1	0.691		GLYMA08G25673.1	0.691
	GLYMA02G10170.1	0.512		GLYMA03G30110.2	0.512

	GLYMA16G05780.1	0.534		GLYMA02G10170.1	0.512
	GLYMA19G26631.1	0.534		GLYMA19G26631.1	0.534
	GLYMA03G30110.2	0.512		GLYMA16G05780.1	0.534
	GLYMA02G29160.3	0.512		GLYMA02G29160.3	0.512

Supplementary Table S7. The interaction protein of GmVLN described.

Gene ID	Protein annotation	Function annotation
GLYMA02G29160.3	Uncharacterized protein; Belongs to the actin family	Cellular process
GLYMA13G41060.1	Uncharacterized protein; Belongs to the actin family	Cellular process
GLYMA15G04360.1	Uncharacterized protein; Belongs to the actin family	Cellular process
GLYMA13G20571.1	Uncharacterized protein LOC100796414 isoform	Acylphosphatase-like domain superfamily
GLYMA02G10170.1	Uncharacterized protein	Actin
GLYMA19G26631.1	Uncharacterized protein; Belongs to the actin family	Organelle organization
GLYMA16G05780.1	Uncharacterized protein	Actin
GLYMA08G25673.1	Uncharacterized protein LOC100500688 isoform	YchF, N-terminal
GLYMA03G30110.2	Uncharacterized protein	Actin
GLYMA04G39380.3	Uncharacterized protein; Belongs to the actin family	Cellular process
GLYMA08G10040.1	Myosin-binding protein 2	Binding
GLYMA05G30150.1	Uncharacterized protein	Zinc finger
GLYMA08G13310.2	Zinc finger transcription factor yy1 isoform x2	Response to acid chemical
GLYMA07G00710.1	Helicase ATP-binding domain-containing protein	Cell cycle
GLYMA07G15710.2	Wuschel-related homeobox 3; Uncharacterized protein	Cell differentiation
GLYMA06G10090.1	Phosphatidylinositol 3-kinase	Membrane protein complex
GLYMA05G27060.2	GTD-binding domain-containing protein	Myosin-binding protein
GLYMA12G31290.1	Uncharacterized protein	Cytoskeletal protein binding
GLYMA09G21600.2	Myosin-binding protein 3 isoform	Myosin binding
LOC547983	Phosphatidylinositol 3-kinase	Vacuole organization
GLYMA16G25580.1	Dirigent protein	Cellular aromatic compound metabolic process
SHAT1-5	Uncharacterized protein	Transcription regulation
GLYMA09G02870.1	Obg-like ATPase 1,Putative uncharacterized protein	GTP binding domain
GLYMA15G13830.1	Putative uncharacterized protein	Response to stress
GLYMA09G06290.1	Uncharacterized protein	Regulation of biological process
GMMYB29B2	MYB/HD-like transcription factor	Response to stress
GLYMA09G06320.1	Uncharacterized protein	Integral component of membrane
GLYMA08G42110.1	Uncharacterized protein	Cellular biosynthetic process
GLYMA19G37000.1	Belongs to the MIP/aquaporin (TC 1.A.8) family	Transport
MYB185	MYB transcription factor MYB185	Tissue development
GLYMA16G02650.2	Auxin response factor	AUX/IAA domain, and auxin binding
GLYMA07G32300.2	Auxin response factor	AUX/IAA domain
GLYMA13G11980.1	Uncharacterized protein	Response to endogenous stimulus
GLYMA07G03030.2	Belongs to the MIP/aquaporin (TC 1.A.8) family	Transport
GLYMA13G32810.1	Mads-box transcription factor, plant	Regulation of biological process
GLYMA08G10550.1	Uncharacterized protein	Transcription regulation

GLYMA13G24240.1	Auxin response factor	Response to hormone
GLYMA14G35030.2	Belongs to the MIP/aquaporin (TC 1.A.8) family	Transmembrane transport

Supplementary Table S8. Protein three-dimensional structure prediction parameters and usage models.

Protein	GMQE	Coverage	Seq Identity	Template
GmVLN1a	0.81	100%	94.71%	I1N029.1.A
GmVLN1b	0.81	100%	100.00%	I1N029.1.A
GmVLN2a	0.77	100%	84.65%	K7K9D0.1.A
GmVLN2b	0.77	100%	84.65%	K7K9D0.1.A
GmVLN3a	0.78	100%	100.00%	K7K9D0.1.A
GmVLN3b	0.78	100%	95.38%	K7K9D0.1.A
GmVLN4a	0.79	100%	82.08%	K7LWU7.1.A
GmVLN4b	0.78	100%	81.77%	K7LWU7.1.A
GmVLN5a	0.79	100%	95.10%	K7LWU7.1.A
GmVLN5b	0.79	100%	100.00%	K7LWU7.1.A

Supplementary Table S9. The raw transcription data of tissues of *GmVLN* Download in LIS (Legume Information System).

Gene ID	Apical Meristem	Flower	Green Pods	Leaves	Nodule	Root Tip	Root
GmVLN1a	4.96	1.6	1.96	0.87	0.69	2.54	1.51
GmVLN1b	6.91	0.8	2.07	0.72	0.42	4.03	2.39
GmVLN2a	9.53	2.01	2.01	1.55	0.2	5.38	1.02
GmVLN2b	14.74	1.82	4.22	1.3	0.75	14.16	2.77
GmVLN3a	6.12	6.82	3.89	5.64	5.51	7.9	5.94
GmVLN3b	9.88	8.74	5.6	7.47	7.65	15.48	11.58
GmVLN4a	4.1	3.75	3.25	2.28	0.82	8.89	3.78
GmVLN4b	6.2	5.01	5.44	3.82	1.5	15.26	4.86
GmVLN5a	0.47	1.09	0	0.89	4.69	0.14	0.06
GmVLN5b	0.24	0.28	0	0.07	0.12	0.07	0

Supplementary Table S10. In q-RT detection, the Ct value of soybean VLN gene in various tissue.

Gene ID	root	stem	leaf	flower	pod	SAM	axillary bud	seed
ACT11 Ct value	16.95	16.39	17.75	19.13	20.65	19.56	19.85	23.18
	16.73	16.44	17.44	19.11	20.44	19.61	19.76	23.29
	16.93	16.43	17.73	19.12	20.34	19.69	19.79	23.3
GmVLN1a Ct value	23.75	22.89	24.67	25.13	25.68	25.63	24.7	30.85
	23.71	22.84	24.78	25.16	25.65	25.64	24.7	30.97
	23.55	22.87	24.78	25.18	25.63	25.3	24.67	30.78
GmVLN1b Ct value	21.47	20.45	23.58	22.91	23.66	24.17	22.54	28.58
	21.46	20.36	23.92	22.93	23.62	24.43	22.54	28.57

	21.56	20.71	23.62	22.87	23.65	24.26	22.55	28.61
GmVLN2a Ct value	24.45	20.63	24.39	24.78	26.2	24.17	23.85	28.91
	24.4	20.62	24.44	24.67	26.17	24.26	23.77	28.84
	24.51	20.78	24.51	24.66	26.32	24.15	23.88	28.8
GmVLN2b Ct value	23.79	20.36	23.86	24.24	25.49	23.64	21.65	22.02
	23.76	20.38	23.88	24.17	25.48	23.76	21.62	22.04
	23.76	20.53	23.65	24.24	25.45	23.72	21.69	22.03
GmVLN3a Ct value	21.51	20.8	22.73	24.55	25.71	25.8	24.72	28.42
	21.49	20.89	22.68	24.44	25.74	25.57	24.67	28.53
	21.65	20.88	22.89	24.49	25.72	25.61	24.78	28.44
GmVLN3b Ct value	21.81	20.97	22.59	23.69	24.77	24.17	23.97	26.8
	21.73	20.94	22.46	23.63	24.7	24.07	23.87	26.86
	21.57	20.96	22.58	23.57	24.6	24.26	23.8	26.79
GmVLN4a Ct value	22.42	20.71	22.24	24.25	25.25	25.07	24.47	28.84
	22.39	20.72	22.48	24.3	25.2	25.09	24.53	28.87
	22.29	20.65	22.38	24.26	25.07	25.07	24.53	28.87
GmVLN4b Ct value	24.58	22.79	23.85	24.3	20.65	25.07	24.01	28.84
	24.59	22.63	23.86	24.26	20.44	25.09	24.47	28.87
	24.61	22.77	23.38	24.42	20.34	25.07	24.53	28.8
GmVLN5a Ct value	30.82	26.94	23.93	26.32	29.06	28.21	24.69	25.3
	30.39	26.97	23.89	26.43	29.01	28.26	24.77	25.33
	30.56	26.71	23.94	26.31	29.09	28.27	24.76	25.24
GmVLN5b Ct value	31.58	27.38	24.67	27.11	29.59	28.93	25.34	25.72
	31.66	27.72	24.78	27.07	29.69	28.89	25.17	25.75
	31.83	27.78	24.78	27.08	29.59	28.89	25.43	25.73
